# Craniometrics Reveal “Two Layers” of Prehistoric Human Dispersal in Eastern Eurasia

**DOI:** 10.1038/s41598-018-35426-z

**Published:** 2019-02-05

**Authors:** Hirofumi Matsumura, Hsiao-chun Hung, Charles Higham, Chi Zhang, Mariko Yamagata, Lan Cuong Nguyen, Zhen Li, Xue-chun Fan, Truman Simanjuntak, Adhi Agus Oktaviana, Jia-ning He, Chung-yu Chen, Chien-kuo Pan, Gang He, Guo-ping Sun, Wei-jin Huang, Xin-wei Li, Xing-tao Wei, Kate Domett, Siân Halcrow, Kim Dung Nguyen, Hoang Hiep Trinh, Chi Hoang Bui, Khanh Trung Kien Nguyen, Andreas Reinecke

**Affiliations:** 1 School of Health Science, Sapporo Medical University, Sapporo, 060-8556 Japan; 2 Department of Archaeology and Natural History, Australian National University, Canberra, ACT 0200 Australia; 3 Department of Anthropology and Archaeology, University of Otago, Dunedin, 9054 New Zealand; 4 School of Archaeology and Museology, Peking University, Beijing, 100871 China; 5 Department of Management, Okayama University of Science, Okayama, 700-0005 Japan; 6 Institute of Archaeology, Vietnam Academy of Social Science, Hanoi, 61 Phan Chu Trinh, Hanoi, Vietnam; 7 Guangxi Institute of Cultural Relic Protection and Archaeology, Nanning, 530003 China; 8 Fujian Museum, Fuzhou, 350001 China; 9 Center for Austronesian Study, Jakarta, 12510 Indonesia; 10 The National Research Center for Archaeology, Jakarta, 12510 Indonesia; 11 Institute of History and Philology, Academia Sinica, Taipei, 11529 Taiwan; 12 Matzu Folk Culture Museum, Nangan, Lienchiang 20942 Taiwan; 13 Institute of Cultural Relics and Archaeology of Hunan, Changsha, 410008 China; 14 Institute of Cultural Relics and Archaeology of Zhejiang, Hangzhou, 310014 China; 15 Hemudu Site Museum, Ningbo, 315414 China; 16 Institute of Archaeology, Chinese Academy of Social Science, Beijing, 100710 China; 17 Henan Provincial Institute of Cultural Heritage and Archaeology, Zhengzhou, 450000 China; 18 Division of Tropical Health and Medicine, College of Medicine and Dentistry, James Cook University, Townsville, Queensland 4811 Australia; 19 Department of Anatomy, University of Otago, Dunedin, 9054 New Zealand; 20 Southern Institute of Social Sciences, Vietnam Academy of Social Science, 49 Nguyen Thi Minh Khai, Ho Chi Minh, Vietnam; 21 Commission for the Archaeology of Noneuropean Cultures of the German Archaeological Institute, 53173 Bonn, Germany

**Keywords:** Archaeology, Biological anthropology

## Abstract

This cranio-morphometric study emphasizes a “two-layer model” for eastern Eurasian anatomically modern human (AMH) populations, based on large datasets of 89 population samples including findings directly from ancient archaeological contexts. Results suggest that an initial “first layer” of AMH had related closely to ancestral Andaman, Australian, Papuan, and Jomon groups who likely entered this region via the Southeast Asian landmass, prior to 65–50 kya. A later “second layer” shared strong cranial affinities with Siberians, implying a Northeast Asian source, evidenced by 9 kya in central China and then followed by expansions of descendant groups into Southeast Asia after 4 kya. These two populations shared limited initial exchange, and the second layer grew at a faster rate and in greater numbers, linked with contexts of farming that may have supported increased population densities. Clear dichotomization between the two layers implies a temporally deep divergence of distinct migration routes for AMH through both southern and northern Eurasia.

## Introduction

Anatomically modern humans (AMH) initially migrated into east Eurasia prior to 65–50 kya^1–6^, yet the details of migration routes and subsequent population histories have been arguable, now clarified through cranio-morphometric studies in coordination with archaeological evidence. Among the most crucial issues to consider, one set of questions pertains to the debates between a Single Wave Model^7^ versus the variants of a Multiple Waves Model^6,8–11^ of AMH radiating outward from Africa, with further implications about how those ancient groups could relate with modern-day populations. Another set of issues has involved the role of farming economies in driving demographic movements and overlays of population histories during the last several thousands of years, wherein the newest cranio-morphometric studies and archaeological findings can point to at least two layers of populations.

Regarding the initial appearance of AMH in east Eurasia, the large-scale cross-regional evidence so far suggests two major groupings, in southern and northern areas, although ultimately they may have derived from a shared ancestry prior to 65–50 kya. On the southern side of east Eurasia, the initial AMH occupants migrated simultaneously into Southeast Asia (SEA) and the ancient Pleistocene continent of Sahul^8,12,13^. On the northern side, the AMH who reached Northeast Asia (NEA) further dispersed into the American continents through the strait of Beringia during the last glacial age^14–17^. These scenarios could be consistent with interpretations of the Single Wave Model or Multiple Waves Model. The picture likely was complicated, granted the growing evidence of numerous localized variations and intermixtures when AMH populations met with Neanderthals and Denisovans^18,19^.

Major influences in population histories can be attributed to the origins and developments of farming societies, involving a number of movements over the course of some thousands of years. Dating at least 9 kya, archaeological investigations have shown how rice and millet farming had emerged first in the Yellow and Yangtze River areas of China, eventually leading to variable outcomes throughout east Eurasia and into Island SEA after 4 kya^20–22^. In parallel with the archaeological evidence, linguistic studies refer to the movements of Austronesian and Austroasiatic language families, linked with contexts of ancient rice and millet farming societies^23–28^.

Given the time depth of the agricultural influence in east Eurasia, the effects in population movements must have been imposed on the pre-existing demography of AMH groups. The details could be remarkably complicated, yet potentially they can be clarified through direct studies of the ancient skeletal remains from the relevant archaeological sites. The pre-farming and post-farming contexts have disclosed objectively different assemblages of artifacts, food remains, house structures, burial practice, and other aspects of material archaeological signatures that may be coordinated with physical anthropological observations such as in cranio-morphometric studies.

## Results

**Figure 1:**
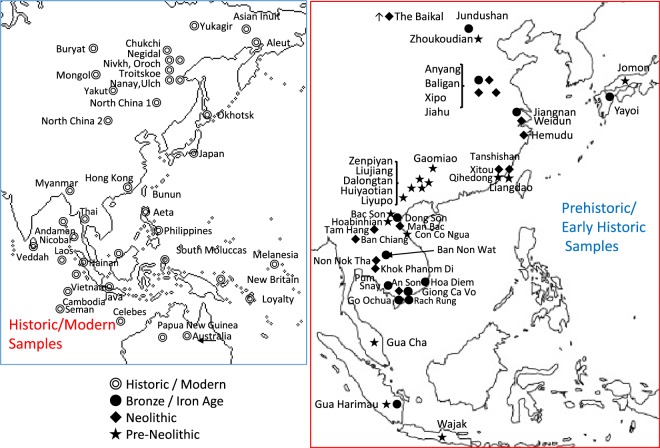
Map showing comparative sample localities.

**Figure 2:**
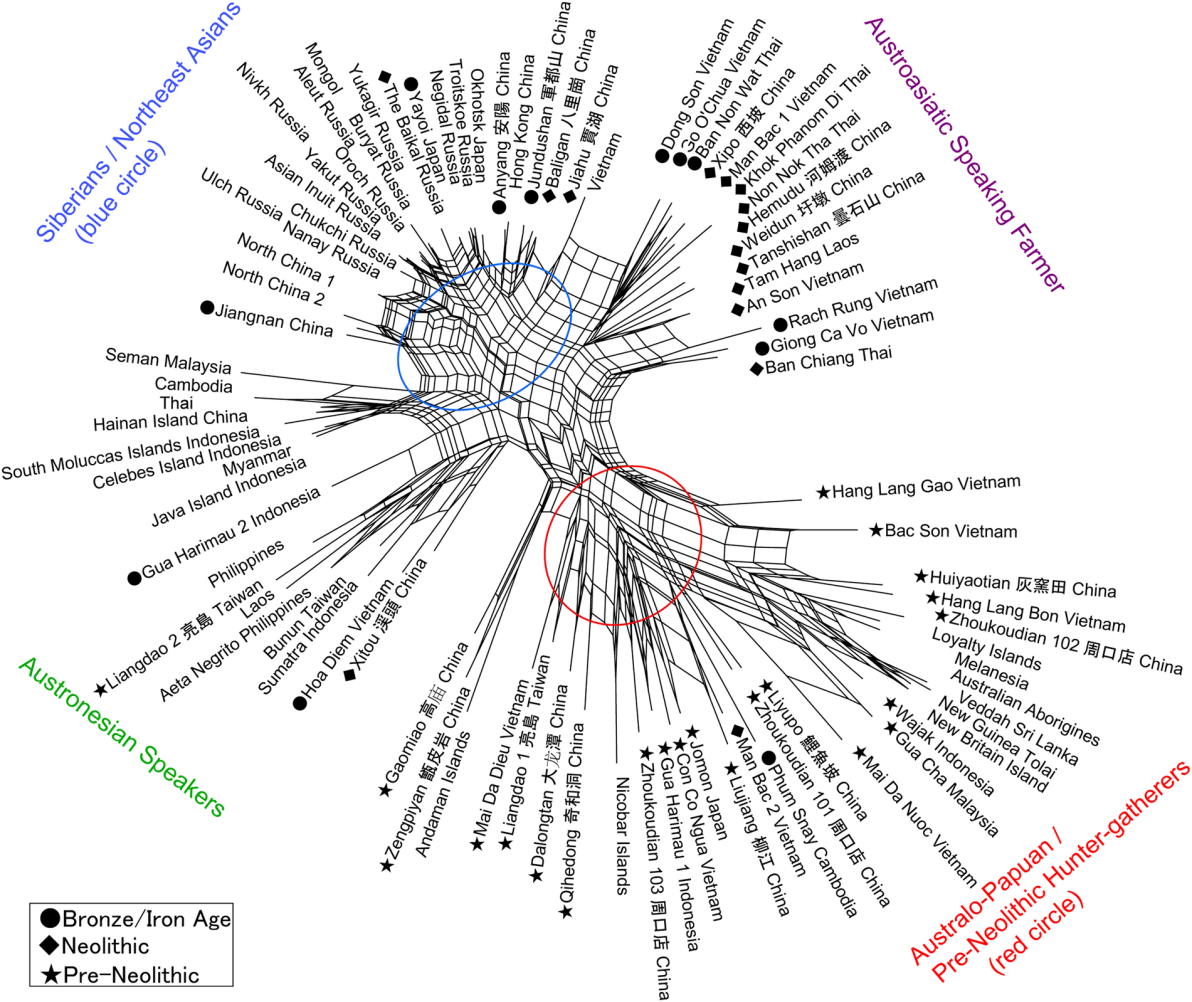
Patterns in cranial affinities. This figure comprises a Phoenix network map that was drawn using Neighbor Net Split analysis based on 16 morphometric measurements.

Two major populations are discerned in the cranial affinities, as expressed through analysis of Q-mode correlation coefficients, based on 16 cranio-morphometric datasets recorded from a total of 89 population samples (Fig. 1, Tables 1 and 2, see Materials and Method section). The results are depicted in a Neighbor Net Split map (Fig. 2), here termed the ‘Phoenix’ tree, due to the shape reminiscent of the mythical bird with large wings.

**Table 1:** Ancient human remains used in this study.

Site/sample	Region	Period	Location	Ref.	Excav.	Remarks	Data source/storageM = Martin’s No.
★ Pre-Neolithic Series							
Zhoukoudian	China	Between ca. 34000 BP and 18000 BP	Beijing City	^29, 30^	—	Individuals nos. 101, 102, and 103	H.M. (cast).
Liujiang	China	Late Pleistocene	Cave site in Liuzhou City, Guangxi Province	^57^	—	Individual no. PA89	H.M. (cast).
Dalongtan	China	ca. 10500 BP	Cave site in Liuzhou City, Guangxi Province	^31^	—	Individual no. 2	H.M./LYZCM
Zengpiyan	China	Between ca. 14000 BP and 10000 BP	Cave site in Guilin City, Guangxi Province	^32^	—	Individuals nos. BT2M1, BT2M4, BT2M5, DT2M1, DT3M1	^32^
Huiyaotian	China	Between ca.9000 BP and 8300 BP	Shell midden in Nanning City, Guangxi Province	^33^	Z.L., H.M., L.C.N., H.C.H., M.Y.	n = 13	H.M./NNGCM
Liyupo	China	Between ca. 7600 BP and 7000 BP	Shell midden in Longan County, Guangxi Province	^33^	Z.L., H.M., L.C.N., H.C.H., M.Y.	n = 6	H.M./NNGCM
Qihedong	China	Between ca. 13000 BP and 9000 BP	Cave site in Zhangping City, Fujian Province	^34^	X.C.F.	Individual no.3	H.M./IVPP
Gaomiao	China	Between 6600 BP and 6400 BP (Beta 328353)	Shell midden in Hongjiang City, Hunan Province	^35^	G.H., H.M., L.C.N., H.C.H.	Individual no. M-02	H.M./HJNGCM
Liangdao 1 (lower layer)	Taiwan Strait	Between 8380 BP and 8204 BP (Beta 321640)	Shell midden at Daowei-I, Liang Island, Matzu	^58^	C.Y.C., C.K.P.	Individual no. LDDW-I-M01	H.M./MFCM
Liangdao 2 (upper layer)	Taiwan Strait	Between 7512 BP and 7374 BP (Beta 336243)	Shell midden at Daowei-I, Liang Island, Matzu	^58^	C.Y.C., C.K.P.	Individual no. LDDW-I-M02	H.M./MFCM
Wajak	Indonesia	Between ca. 37400 BP and 28500 BP	Java Island	^59, 60^	—	Individual no. 2	H.M. (cast).
Gua Harimau 1 (lower layer)	Indonesia	Between ca. 5600 BP and 4400 BP	Cave site in Sumatra	^40, 61^	T.S., A.A.O., H.M., L.C.N., M.O., A.W., H.C.H.	Averages of two Individuals no. 74 and no. 79	H.M./UPTDM^40^
Gua Cha	Malaysia	Hoabinhian, Between ca. 8000 BP and 6000 BP	Cave site in Kelantan Province	^62^	—	Individual no. H12	H.M./LCHES
Hang Lang Gao	Vietnam	Hoabinhian	Cave site in Hoa Binh Province	^63^	—	Averages of two individuals no. 17 and no. 19	H.M./MNHN
Hang Lang Bon	Vietnam	Hoabinhian	Cave site in Thanh Hoa Province	^64, 65^	—	Individual (no number)	H.M./MNHN
Mai Da Dieu	Vietnam	Epi-Hoabinhian	Cave site in Thanh Hoa Province	^66^	L.C.N.	Individual no. 86MMD-M16 M17 is estimated	H.M./VKCH
Mai Da Nuoc	Vietnam	Hoabinhian, ca. 8000 BP	Cave site in Thanh Hoa Province	^66^	L.C.N.	Individual no. 84MDN-M1	H.M./VKCH
Bac Son	Vietnam	Epi-Hoabinhian, between ca. 8000 BP and 7000 BP	Cave sites in Pho Binh Gia, Cua Git, Lang Cuom, and Dong Thuoc	^67^	—	n = 7	H.M./MNHN
Con Co Ngua	Vietnam	Da But Culture, between ca. 6700 BP and 6200 BP	Shell midden in Thanh Hoa Province	^39, 68^	M.O., H.H.T., A.W., K.D, L.C.N., H.M.	n = 36	H.M./VKCH
Jomon	Japan	Middle-latest Phase between ca. 5000 BP and 2300 BP	Known from across the whole of Japan	^69^	—		^71, 107^
♦ Neolithic Series							
Xitou	China	Tanshishan Culture, between ca. 5000 BP and 4300 BP	Fujian Province	^72, 73^	X.C.F.	n = 7	H.M./FJNGPM
Tanshishan	China	Tanshishan Culture, between ca. 5000 BP and 4300 BP	Fujian Province	^74^	X.C.F.	n = 4	H.M./FJNGPM
Hemudu	China	Hemudu Culture, between ca. 7000 BP and 5300 BP	Zhejiang Province	^75^	G.P.S., W.J.H.	Individual no. M23	H.M./HEMSM
Baligan	China	Yansgao Culture, between ca. 7000 BP and 5000 BP	Henan Province	^76^	C.Z., J.N.H.	n = 26	H.M. /PKU
Xipo	China	Yansgao Culture, ca. 5300 BP and 5000 BP	Henan Province	^77^	X.W.L.	n = 11	H.M./HEMSM
Jiahu	China	Jiahu Phase 1, between ca. 9000 BP and 8000 BP	Henan Province	^78, 79^	X.T.W.	Individual no. M395	H.M./HPICHA
Weidun	China	Majiabang Culture, between ca. 7000 BP and 6000 BP	Jiangsu Province	^80^	—		^80^
Tam Hang	Laos	ca. 3500 BP	Hua Pan Province	^81, 82^	—	Averages of two individuals no. S10 and no. S14	H.M./MNHN
Ban Chiang	Thailand	Neolithic-Bronze Age, between ca. 4100 BP and 2300 BP	Site in Udon Thani Province	^83, 84^	—		^84^; M43(1), 43c, 46b, 46c, 57, 57a by H.M./UHW, SAC (n = 15)
Khok Phanom Di	Thailand	Between ca. 3800 BP and 3500 BP	Site in Chonburi Province	^85, 86^	C.H., A.K., N.T.	n = 19	^86^; M43(1), 43c, 46b, 46c, 51, 52, 57,57a by H.M./FAD
Non Nok Tha	Thailand	Neolithic-Bronze Age, between ca. 3500 BP and 2500 BP	Site in Khok Kaen Province	^87^	—	n = 22	H.M./UNLV
Man Bac 1	Vietnam	Between ca. 3800 BP and 3500 BP	Ninh Binh Province (indigenous group)	^88, 89^	H.M., M.O., K.D.N., M.Y., L.C.N., H.H.T., K.D.	n = 5	H.M./VKCH published ^88^
Man Bac 2 (outlier)	Vietnam	Between ca. 3800 BP and 3500 BP	Ninh Binh Province (immigrant group)	^88, 89^	H.M., M.O., K.D.N., M.Y., L.C.N., H.H.T, K.D.	n = 12	H.M./VKCH published ^88^
An Son	Vietnam	ca. 3800 BP	Long An Province	^90, 112^	C.H.B., L.C.N.	n = 4 sample used 2004 series	H.M./LAPM
Baikal	Russia	Neolithic		^91^	—		^111^
*•Bronze - Iron Age*							
Anyang	China	Late Shang Dynasty, between ca. 3300 BP and 2996 BP (1350-1046 BC)	Henan Province	^92^	—		^92^ M43(1), 43c, 46b, 46c, 57, 57a by H.M./SINICA (n = 26)
Jiangnan	China	Zhou - Han Dynasty, between 2720 BP and 1730 BP (770 BC- AD 220)	Sites along the Lower Yangtze River	^93^	—		^93^
Jundushan	China	Spring and Autumn Period, ca.2720-2353 BP (770-403 BC)	Beijing City	^94^	J.N.H.	n = 27	H.M./PKU
Gua Harimau 2 (upper layer)	Indonesia	Between ca. 2400 BP and 1700 BP	Cave site in Sumatra Island	^40, 61^	T.S., A.A.O., H.M., L.C.N., M.O., A.W., H.C.H.	n = 10	H.M./UPTDM^40^
Ban Non Wat	Thailand	Bronze-Iron Age, between ca. 3100 BP and 1400 BP	Nakhon Ratchasima Province	^95– 98^	C.H., R.T., A.K., N.T., S.H.	n = 29 (combined crania no. 86 and no. 566)	H.M./FAD
Phum Snay	Thailand	Iron Age, between ca. 2350 BP and 1800 BP	Banteay Meanchey Province	^99, 100^	D.O., K.D., S.H.	n = 33	H.M./RUFA, WB&WL published^100^
Dong Son	Vietnam	Dong Son Period, between ca. 2500 BP and 1700 BP	Thanh Hoa Province	^101^	L.C.N.		^101^; M43(1), 43c, 46b, 46c, 57, 57a by H.M./VKCH, CSPH (n = 20)
Giong Ca Vo	Vietnam	Iron Age, between ca. 2500 BP and 2000 BP	Can Gio District, Ho Chi Minh City	^102, 112^	L.C.N., K.D.N., C.H.B.		^112^; M43(1),43c,46b,46c, 57,57a by H.M./HCMHM (n = 4)
Go O Chua	Vietnam	Iron Age, human remains between ca. 2400 BP and 2100 BP	Long An Province	^103, 104^	A.R., L.C.N.	LAPM	L.C.N. in press/LAPM
Hoa Diem	Vietnam	Iron Age, between ca. 1900 BP and 1800 BP	Khanh Hoa Province	^105^	M.Y., C.H.B., K.T.K.N., K.D.N., H.M., L.C.N.	n = 6	H.M./KHPM (n = 6)
Rach Rung	Vietnam	Bronze Age, ca. 2800 BP	Long An Province	^106^	L.C.N.	Individuals no. MH1 and no. MH3	H.M./LAPM (n = 2)
Yayoi	Japan	Yayoi Period, between ca. 2800 BP and 1700 BP	Doigahama, Kanenokuma and other immigrant sites in Northern Kyushu and Yamaguchi districts	^70^			^70^

Abbreviations: n, sample size; Ref., reference (number in squared brackets, e.g. [No.]); Excav., recent excavation contributor including for post excavation work among current first authors and co-authors, and researchers in acknowledgements (in alphabetical order); C.H.B., Chi Hoang Bui; C.Y.C., Chung-yu Chen; K.D., Kate Domett; X.C.F., Xue-chun Fan; S.H., Siân Halcrow; G.H., Gang He; J.N.H., Jia-ning He; C.H., Charles Higham; W.J.H., Wei-jin Huang; H.C.H., Hsiao-chun Hung; X.W.L., Xin-wei Li; Z.L., Zhen Li; H.M., Hirofumi Matsumura; K.D.N., Kim Dung Nguyen; L.C.N., Lan Cuong Nguyen; K.T.K.N., Khanh Trung Kien Nguyen; D.O., Dougald O’Reilly; A.A.O., Adhi Agus Oktaviana; M.O., Marc Oxenham; C.K.P., Chien-kuo Pan; A.R., Andreas Reinecke; G.P.S., Guo-ping Sun; T.S., Truman Simanjuntak; H.H.T., Hoang Hiep Trinh; R.T., Rachanie Thosarat; X.T.W. Xing-tao Wei; A.W., Anna Willis; M.Y., Mariko Yamagata; C.Z., Chi Zhang. Data sources: H.M., measured by Hirofumi Matsumura. Repositories (for materials measured by H.M.): BNHM, Department of Palaeontology, British Natural History Museum, London, UK; CSPH, Center for Southeast Asian Prehistory, Hanoi, Vietnam; FAD, Fine Arts Department, Pimai, Thailand; FJNGPM, Fujian Museum, Fujian, China; HCMHM, Ho Chi Minh Historical Museum, Ho Chi Minh, Vietnam; HEMSM, Hemudu Site Museum, Zhejiang, China; HJNGCM, Hongiang City Museum, Hunan, China; HPICHA, Henan Provincial Institute of Cultural Heritage and Archaeology, Zhengzhou, China; IVPP, Institute of Vertebrate Paleontology and Paleoanthropology, Chinese Academy of Sciences, Beijing, China; KHPM, Khanh Hoa Provincial Museum, Nha Trang, Vietnam; LAPM, Long An Provincial Museum, Vietnam; LCHES, Leverhulme Centre for Human Evolutionary Studies, University of Cambridge, UK; LYZCM, Liuzhou City Museum, Guangxi, China; MFCM, Matzu  Folk Culture Museum, Lienchiang, Taiwan; MNHN, Laboratoire d’Anthropologie Biologique, Musée de l’Homme, Paris, France; NMP, National Museum of the Philippines, Manila, Philippines; NNGCM, Nanning City Museum, Guangxi, China; NTU, College of Medicine, National Taiwan University, Taipei, Taiwan; PKU, School of Archaeology and Museology, Peking University, Beijing, China; RUFA, Royal University of Fine Arts, Phnom Penh, Thailand; SAC, Princess Maha Chakri Sirindhorn Anthropology Centre, Bangkok, Thailand; SINICA, Academia Sinica, Taipei, Taiwan; UHW, Department of Anthropology, University of Hawaii, USA; UNLV, Department of Anthropology, University of Nevada, Las Vegas, USA; UPTDM, Museum, Sipatad Lidah, Sumatra, Indonesia; USYD, Department of Anatomy, University of Sydney, Australia; VKCH, The Vietnam Institute of Archeology (Vien Khao Co Hoc), Hanoi, Vietnam; WB&WL, Temples of Wat Bo and Wat Leu, Cambodia.

**Table 2:** Modern population samples used in this study.

Population/region	Data set 1 ([No.] = reference)	Data set 2 ([No.] = reference)	Remark (M = Martin’s number)	Repository
Aeta Negrito/Philippines	H.M. (n = 11)	H.M. (n = 11)	—	MNHN
Andaman Islands	^52^	H.M. (n = 5)	M9, 51 by H.M. (n = 22)	BNHM, LCHES
Aborigines/Australia	^71^	H.M. (n = 21)	—	BNHM
Bunun/Taiwan	^54^	H.M. (n = 16)	M45, 48, 51, 55 by H.M. (n = 22)	NTU
Cambodia	H.M. (n = 12)	H.M. (n = 12)	—	MNHN
Celebes Island/Indonesia	^53^	^107^	M17, 45, 48, 51 by H.M. (n = 6)	BNHM
Hainan Island/China	^52^	H.M. (n = 24)	M48, 51, 55 by H.M. (n = 24)	NTU
Hong Kong/China	H.M. (n = 7)	H.M. (n = 7)	—	LCHES
Japan	^72^	^107^	—	—
Java Island/Indonesia	^53^	^107^	M17, 45, 48, 51 by H.M. (n = 20)	BNHM, LCHES
Laos	^112^	H.M. (n = 10)	—	MNHN
Loyalty Islands	H.M. (n = 17)	H.M. (n = 18)	—	MNHN
Melanesia	^71^	^107^	Fiji, Tonga; New Hebrides; New Guinea	—
Myanmar	^53^	^107^	M17, 45, 48, 51 by H.M. (n = 20)	BNHM
New Britain Island	H.M. (n = 20)	H.M. (n = 19)	—	LCHES
Tolai/New Guinea	^52^	H.M. (n = 26)	M9,48,51 by H.M. (n = 20)	USYD, LCHES
Nicobar Islands	H.M. (n = 13)	H.M. (n = 9)	—	LCHES
Northern China 1	^71^	^107^	Jiling Province	—
Northern China 2	^71^	^107^	Manchuria Province	—
Philippines	^108^	H.M. (n = 8)	—	NMP
Seman/Malaysia	H.M. (n = 1)	H.M. (n = 1)		BNHM
South Moluccas Islands/Indonesia	^53^	^107^	M17, 45, 48, 51 by H.M. (n = 4)	BNHM
Sumatra Island/Indonesia	^53^	^107^	M17, 45, 48, 51 by H.M. (n = 8)	BNHM, LCHES
Thai	^109^	^107^	—	—
Veddah/Sri Lanka	H.M. (n = 2)	H.M. (n = 2)	—	LCHES
Vietnam	H.M. (n = 27)	H.M. (n = 27)	—	MNHN
Okhotsk/Japan	^110^	^110^	Between ca. 1600 BP and 1000 BP in Hokkaido, Japan	—
Asian Inuits, Aleut, Buryat, Chukchi, Mongol, Nanay, Negidal, Nivkh, Oroch, Troitskoe, Ulch, Yakut, Yukagir (Russia)	^111^	^111^		—

Abbreviations (as in footnote  of Table 1): n, sample size; Data set 1: M1, maximum cranial length; M8, maximum cranial breadth; M9, minimum frontal breadth; M17, basion-bregma height; M45, bi-zygomatic breadth; M48, upper facial height; M51, orbital breadth; M52, orbital height; M54, nasal breadth; M55, nasal height. Data set 2: M43(1), frontal chord (FC); M43c, frontal subtense (FS), M57, simotic chord (SC); M57a, simotic subtense (SS); M46b, zygomaxillary chord (ZC); M46c, zygomaxillary subtense (ZS). Repositories are listed here only for materials measured by H.M.

The ‘Phoenix’ tree shows a straightforward dichotomization in two major clusters. (1) The ‘head’ cluster (upper left side) includes Northeast and East Asians (blue circle), as well as Southeast Asians, for the most part referring to early farming and later populations. (2). The ‘tail’ (lower right side) cluster includes Australo–Papuans and late Pleistocene/early Holocene East/Southeast Asians (red circle), strongly corresponding with pre-farming and Hoabinhian contexts.

Within the overall clustering patterns, naturally some overlap or exchange can be seen in a closer examination, as an expected outcome of small-scale admixture. For example, the data points for Austroasiatic-speaking farmers are branched from the East Asian cluster, slightly toward the side of the red circle cluster that primarily would refer to Australo-Papuan groups. Similarly, the sub-cluster for Austronesian-speaking groups in Island SEA deviates somewhat from the East Asian cluster and instead branches toward the Australo-Papuan affinity. Deviating from the Australo-Papuan cluster, a few samples such as from Gaomiao, Zengpiyan, and the Andaman Islands appear to share a slight affinity with the NEA populations.

## Discussion

If the original AMH populations across eastern Eurasia during the Pleistocene possessed mostly Australo-Papuan affinities, then how and when did these groups diminish while distinct East Asian affinities became widespread more recently? In order to address this issue, we examined a series of human skeletal remains from archaeological sites in China, Japan, Russia, and Southeast Asia that derive from multiple pre-farming, early farming, and later contexts of the Late Pleistocene through AD 300.

Human skeletal remains and fossils sites of the last major ice age (Pleistocene) were crucial in this research. A range of Late Pleistocene crania from the Upper Cave at Zhoukoudian (northern China), as well as from sites at Liujiang (southern China), Minatogawa and Shiraho-Saonetabaru (Japan), Tam Pa Ling (Laos), Moh Khiew (Thailand), Tabon (Philippines), Niah (Malaysia), Wajak (Indonesia) and others, have been dated within the range of 47 kya to 16 kya^3,20^. Preservation of measurable characteristics was a major concern, yet in total these specimens supplied multiple confident cranio-morphometric measurements.

The Phoenix map reveals close cranial affinities between the archaeological samples from the Upper Cave at Zhoukoudian and those from the Liujiang and Wajak sites, as well as with the larger Australo-Papuan and Veddha-Andaman groupings. This result suggests that the Late Pleistocene people who lived at these sites shared genetic ancestry with AMH settlers across much of eastern Eurasia, including as far to the east as modern-day Australia and New Guinea. Dispersal of AMH at this time coincided with Pleistocene glacial conditions when significantly lower global sea levels had created vast land masses and shorter water crossings from East Asia through Mainland and Island SEA and as far as Australia and New Guinea.

We documented a continuation of the “first layer” AMH in southern China on the basis of hunter-gatherer sites that were dated between ca. 14 kya and 5 kya (Fig. 2). These study sites included Dalongtan, Zengpiyan, Huiyaotian, and Liyupo in Guangxi Province, Gaomiao in Hunan Province, Qihedong in Fujian Province, and Liangdao in the Taiwan Strait. Although some site contexts within this group chronologically coincided with the earliest known rice and millet farming in Yellow and Yangtze River regions, hunter-gatherer groups still had occupied southern areas. From those hunter-gatherer sites, diagnostic features of skeletal remains included the presence of dolichocephalic calvaria, large zygomatic bones, remarkably prominent glabellae and superciliary arches, concave nasal roots, and low and wide faces^1,29–35^. Notably, ancient Japanese Jomon hunter-gatherers belonged to this same grouping.

In addition to the samples from China, pre-Neolithic SEA hunter-gatherer groups were represented in this analysis mostly by archaeological samples from cave sites that contained pebble-tool complex of “Hoabinhian” associations^36–38^. Our Phoenix map (Fig. 2) reveals that all of the analyzed Hoabinhian remains from Vietnam and Malaysia shared cranial characteristics with Australo-Papuans. These traits were retained into later post-Hoabinhian hunter-gatherer contexts, including the shell midden site of Con Co Ngua (Vietnam), dated around 6.5 kya^39^. Likewise, the remains of hunter-gatherers recovered from the ca. 5 kya Gua Harimau site (Sumatra, Indonesia) share close affinities with Australo-Papuans^40^.

The “second layer” population identified in this study is associated with present-day NEA people, including all Siberian ethnic groups. The tight clustering of cranial morphologies reflects strong inter-group homogeneity that can be explained most parsimoniously via the single shared origin of a flat and long face and comparatively short head. These definitive characteristics may have originated among people who lived in cold conditions and adapted by reducing their total body surface.

The early hunter-gatherer communities gave way to populations with northern morphometric affinities, seen at Neolithic and Bronze-Iron Age population samples in eastern Eurasia. The prevailing hypothesis for the origin of the “second layer” and the spread of its descendants across much of East Asia and SEA implies a key role for rice and millet agriculture in China in promoting population growth and expansion. Such farming traditions now are traced confidently to 9 kya within the Yellow and Yangtze River area^21–23^. Between 7 kya and 5 kya, rice and millet agriculture supported a number of large settlements encompassing an expanding geographical range across China, and several of the resident groups developed complex social, political, economic, and religious systems^22,23^.

The early Chinese farming groups represented here from the archaeological sites of Jiahu, Baligan, Xipo (Henan Province), Hemudu, Weidun (Zhejiang Province), Xitou, and Tanshishan (Fujian Province) all exhibit close affinities with their NEA Siberian counterparts (Fig. 2). With these results, we infer that the “second layer” of population was associated with the earliest occurrences of farming in this region. Moreover, we interpret that the “second layer” of population had been affected by NEA-associated gene flow from the north, demonstrably differentiated from pre-existing Australo-Papuan traits seen in our older Chinese and SEA samples.

Previous research utilizing archaeological evidence and language history has demonstrated that a remarkable cultural transition took place in SEA between 4.5 and 4 kya^24–27^. This conclusion now is reinforced by the “second layer” identified here on the basis of skeletal remains, specifically from the sites of Man Bac and An Son (Vietnam), Tam Hang (Laos), and Ban Chiang, Khok Phanom Di, Ban Non Wat, and Non Nok Tha (Thailand) (Fig. 2). This cross-regional archaeological signature reflects the geographic expansion of a “Neolithic” horizon of advanced pottery and stone tool traditions, farming economies, and residential settlement structures that can be traced ultimately to the Yangtze River Valley (e.g. Hemudu in Zhejiang Province in Fig. 2) before it had spread through southern China, Mainland and Island SEA, Taiwan, and eventually into Pacific Oceania.

The cranio-morphological datasets in this study consistently confirm affinities with NEA-derived “second layer” populations at “Neolithic” sites dated between 5 kya and 4 kya in southern China and SEA and slightly later in Oceania. This relationship is corroborated by the fact that burial traditions at these locations involved extended-position interment, in contrast to the older flexed-position formats^39,41^. The findings are most striking in China and SEA, where archaeological records reveal “first layer” affinities for thousands of years in duration that suddenly were replaced across large geographic scales by groups of the “second layer”.

The interface between different populations appears to have been more complex in some regions of SEA where the “first layer” occupants were well established and somewhat diversified for long periods of time before the notably late arrivals of the “second layer” after 2.4 kya. Such late transitions during Bronze or Iron Ages occurred at Hoa Diem (Vietnam) and in the upper layer at Gua Harimau (Sumatra, Indonesia) (Fig. 2). Those close cranial affinities with Bunun (Taiwan), Sumatra, and the Moluccas, Philippines, and Celebes Islanders suggest vigorous human movements, trade networks, and other exchanges crossing through South China. Besides, their clustering with the Neolithic Xitou (Fujian Province) in southern China, later arrival Liangdao 2 (Matzu Island in the Taiwan Strait) implies their remote homeland somewhere in southern China. In Liangdao and Gua Harimau (see: Liangdao 1, Gua Harimau 1 in Fig. 2), provide an extensive evidence for a replacement between local indigenous populations with extremely deep prehistorical roots from Pleistocene, and secondary movements of migrants from the north across Southern China Sea. Linguistic evidence^28^ equates these ancient expansions with the Austronesian language family in Taiwan, parts of Mainland SEA, most of Island SEA, and into Oceania, as well as with the Austroasiatic language family in Mainland SEA.

The overall heterogeneity seen in this sub-cluster suggests regional variations in the degrees of genetic admixture between first and second layer populations, although the NEA features are very dominant. The clustering with Non-Austronesian groups (Fig. 2, Thai, Myanmar, Cambodia, and Laos) can be explained by the results of variable intermixture ratios between the descendants of early indigenous groups and later immigrants. Such a patterning cannot be reconciled with a single origin or regional continuity model of all populations in total. Aeta and Semang Negritos, despite possesing phenotypically different features from surrounding people (small body size and dark skin color), in particular appear to have interbred with the surrounding populations. Given the greater heterogeneity among SEA samples, in contrast to the homogeneity of NEA samples, the most probable scenario had involved a strongly homogeneous genetic input from NEA population flows into the diverse SEA region.

Our data reveal a clear dichotomy between first and second population layers that remains consistent across large geographic scales and implies a shared genetic origin for the emergence of the second layer as well as its spread across eastern Eurasia. This degree of cross-regional consistency points to a strongly unified “second layer” of population, rather than the much less likely coincidental convergence of the same outcome at multiple sites due to the effects of climate, diet, nutrition, or other localized factors that might have influenced cranial morphology.

These findings from cranial measurements find extra support from non-metric dental morphology^42^, generally believed under strong genetic control and free of environmental influence, pointing to the same two layers of populations. One grouping is apparent in Australo-Papuan and early SEA teeth, consistent with the “first layer”. Another grouping is apparent in NEA and American natives, consistent with the “second layer.” Future research may consider the deeper relation between NEA and American populations, likely involving a shared ancestry through Siberia during the Pleistocene.

Our findings are congruent with the emerging picture in genome data^43–46^. One key point has been a deep population divergence in AMH, suggesting a branching event prior to the diversification of present-day east Eurasian populations. Traces of such a deep divergence were found in samples from Vietnam, Philippine Negritos, and Jomon hunter-gatherers in Japan. The genome study^45^ found Denisova admixture most notably in the Philippine Negritos and to variable extent in other Australo-Papuan populations, thus indicating a distinctive ancient contribution in the “first layer” of the SEA landmass and extending into the ancient Pleistocene continent of Sahul. Meanwhile, Denisova ancestry was absent in mainland Asian populations^18^. The missing Denisova genome in NEA suggests a separate origin from the SEA and Sahul occupants who linked with modern-day Australo-Papuans and Philippine Negritos.

In terms of the deeper origins of the apparently homogenous NEA population, we may consider the more ancient homelands and migratory routes, prior to the entrance into the Yellow and Yangtze River areas by 9 kya but potentially much earlier. In one possible scenario, ancient people perhaps of the “first layer” with Australo-Papuan features moved into Siberia and subsequently adapted to the extremely cold climate during the Last Glacial Maximum (LGM) of 24– 16 kya. Another possibility may have involved a Western Asia or European origin, wherein people migrated from western to eastern Siberia across northern Eurasia. In any case, this issue is unresolved, because the ancestral morphology of NEA people so far has been undefined in the scarce skeletal material from the Pleistocene of Siberia. In the Siberian regional samples, so far not enough cranial measurements can refer to the ancient periods pre-dating the cold climate adaptations such as facial flattening. Until these and other issues can be resolved, our study cannot expand to compare substantively with similar-age cranial data from the western hemisphere.

Among the few known pre-40 kya Siberian AMH samples, the DNA analysis of the Ust’-Ishim specimen dated to 45 kya offered a high-quality genome sequence^19^, wherein this AMH individual derived from the basal population of northern Eurasia. This individual had shared ancestors in common with present-day east Eurasian populations and pre-farming west Eurasian populations, with a trace of Neanderthal gene. Another DNA analysis has been possible with the 40 kya AMH in Tianyuan Cave near Beijing^47^, revealing a close genetic relationship with present-day East Asians and evidently different from the diagnostic DNA markers in current European people, therefore suggesting a divergence between European and Asian populations at least in this case. Interpretations may yet be modified with future findings in more cross-regional samples from these ancient time frames.

Taking all of the evidence into consideration, two populations of AMH in eastern Eurasia reflect a deep divergence that most likely accorded with separate migrating events and routes, as expressed in our “two layer” model. The two migration contexts may have been separated by the natural boundary of the Himalaya mountainous zone, posing a barrier between southern and northern routes. The southern route would concur with views of AMH following the coastal rim of the Indian sub-continent and continuing through SEA and onward into the ancient Pleistocene continent of Sahul, at least as early as 50 kya, linked with the later emergence of the archaeologically defined Hoabinhian stone tool complex of this region. So far, the northernmost trace of this “first layer” group has been verified in the Zhoukoudian Upper Cave series in our craniometrics analysis.

**Figure 3:**
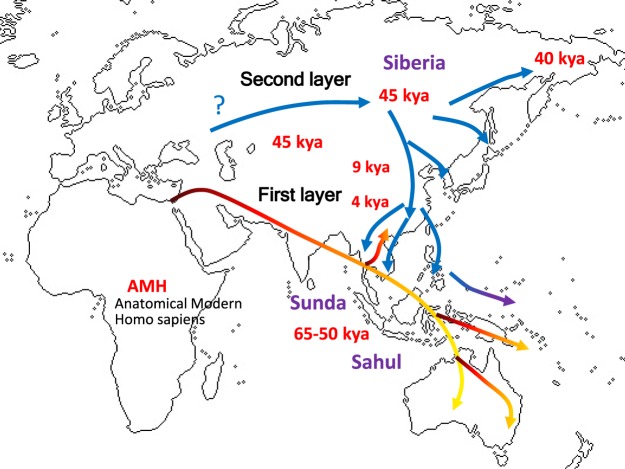
Two layer model of anatomically modern human (AMH) population movements across eastern Eurasia.

The northern route of AMH is less clear in terms of paleo-geographic mapping. In our hypothetical scenario, the NEA ancestral groups had migrated across Siberia from western Eurasia around 45 kya^1,4,19^, and their archaeological signatures involved microblade traditions^23^. Their descendants later developed a quite different cultural trajectory in China after 9 kya, with domesticated millet and rice, and their even later descendants expanded to occupy the larger region while bringing variations of farming economies with them (Fig. 3).

Our “two layer” model in eastern Eurasia may contribute to discussions of global-scale population dispersals and interactions. Our findings are compatible with the AMH dispersal model in west Eurasia, advocated by genome data^48^. Most present-day Europeans derived from later arrivals, along with farming dispersals from the Near East, admixed with a pre-existing base of indigenous hunter-gatherer Eurasian population.

The results of this study are congruent with the archaeological signature of a geographic expansion of “Neolithic” groups, as an added layer flowing through pre-existing populations. In our particular study case, the “second layer” groups can be defined not only by their cranio-morphometric features but also by their pottery traditions, extended-position burials, residential settlements, and farming economies. These groups brought Austroasiatic languages to the mainland and Austronesian languages to the islands from Taiwan southward. Independently confirming our interpretation, other studies of ancient genome analysis^43,44,49^ and nonmetric dental traits^42,50^ have demonstrated the rapid contribution of NEA genes into SEA, explained by large-scale population expansions of farming groups.

## Materials and Methods

The materials used in this study are archaeological and modern cranial series from Northeast Asia, Southeast Asia, and Pacific Oceania, listed in Tables 1 and 2, and all localities are summarized in Fig. 1. The dataset includes samples from contexts of Late Pleistocene, Early to Middle Holocene, Neolithic, Bronze Age, Iron Age, Proto-Historic, Historic, and Modern. Space precludes a review of each sample in the dataset, while the references in Table 1 provide details of the majority of the primary sources. The chronological category ‘Neolithic’ is assigned to communities with clear evidence for agricultural subsistence without metal, regardless of pottery manufacture, according to the current professional standards in this region^26,39^.

Geographic terminology is of crucial importance in this study. “Northeast Asia (NEA)” encompasses modern China, North and South Korea, Japan, Mongolia, and the Russian Far East including Sibera. “Southeast Asia (SEA)” refers to modern Myanmar (Burma), Thailand, Vietnam, Laos, Cambodia, Malaysia, Singapore, Indonesia, Brunei, the Philippines, Taiwan and the Andaman and Nicobar Islands. “Eastern Eurasia” refers the area encompassing to both the NEA and SEA.

In total, 16 cranial measurements from male samples were utilized, based on the definitions of Martin^51^: M1 = maximum cranial length, M8 = maximum cranial breadth, M9 = minimum frontal breadth, M17 = basion-bregma height, M45 = bi-zygomatic breadth, M48 = upper facial height, M51 = orbital breadth, M52 = orbital height, M54 = nasal breadth, M55 = nasal height, M43(1) = frontal chord (FC), M43c = frontal subtense (FS), M57 = simotic chord (SC), M57a = simotic subtense (SS), M46b = zygomaxillary chord (ZC), M46c = zygomaxillary subtense (ZS). These measurements were obtainable for cranial affinity including both the calvaria and facial profiles, and they were the most consistently available measurements among the comparative samples. Approximately 800 skeletons were measured by the first author H.M, augmented by documented data from other researchers if possible. In addition to the citation data in Tables 1 and 2, raw data and group averages of cranial measurements are given in the separate files of Tables S1 and S2.

Among the cited data, discrepancies are evident in the measurement systems of upper facial height and orbital breadth between Howell’s data^52^ and the procedures of other researchers. Howell’s upper facial height (NPH) was measured at the anatomical point of the prosthion, while others used the alveolar point according to Martin’s method (M48). As for the orbital breadth, Howell used the dacryon (OBB), while most others used the maxillofrontale (M51). Pietrusewsky^53,54^ (cited in Tables 1 and 2) adopted Howell’s method for these measurements. For those incompatible measurements, as well as missing measurement items, data were recorded in the present study in accordance with the first authors using applicable cranial specimens (see: data source in Table 1, remark in Table 2).

The craniometric affinities of comparative samples were assessed with Q-mode correlation coefficients^55^, using the standardized 16 measurements of group averages. Then standard deviation data was used from the Thai sample which provides the largest sample size among the comparative groups. Concerning the ancient archaeological samples in this study, the available data often required working with single specimens as representative of their sites, especially for those sites of late Pleistocene and early Holocene contexts. So far, the cranial affinity can be assessed at the individual level in most cases. As this study does not rely on statistical significance tests, the potential error is negligible in the use of averaged data.

In order to aid our interpretation of phenotypic affinities between the samples, Neighbor Net Split tree diagrams were generated using the software Splits Tree Version 4.0^56^, applied to the distance (1-r) matrix of Q-mode correlation coefficients (r) in a separate file (Table S3).

## Electronic supplementary material

Table S1

Table S2

Table S3

## Data Availability

Supplementary data are available in the online version of this work.
