# Author Correction: Craniometrics Reveal “Two Layers” of Prehistoric Human Dispersal in Eastern Eurasia

**DOI:** 10.1038/s41598-019-44355-4

**Published:** 2019-05-23

**Authors:** Hirofumi Matsumura, Hsiao-chun Hung, Charles Higham, Chi Zhang, Mariko Yamagata, Lan Cuong Nguyen, Zhen Li, Xue-chun Fan, Truman Simanjuntak, Adhi Agus Oktaviana, Jia-ning He, Chung-yu Chen, Chien-kuo Pan, Gang He, Guo-ping Sun, Wei-jin Huang, Xin-wei Li, Xing-tao Wei, Kate Domett, Siân Halcrow, Kim Dung Nguyen, Hoang Hiep Trinh, Chi Hoang Bui, Khanh Trung Kien Nguyen, Andreas Reinecke

**Affiliations:** 1 School of Health Science, Sapporo Medical University, Sapporo, 060-8556 Japan; 2 Department of Archaeology and Natural History, Australian National University, Canberra, ACT 0200 Australia; 3 Department of Anthropology and Archaeology, University of Otago, Dunedin, 9054 New Zealand; 4 School of Archaeology and Museology, Peking University, Beijing, 100871 China; 5 Department of Management, Okayama University of Science, Okayama, 700-0005 Japan; 6 Institute of Archaeology, Vietnam Academy of Social Science, Hanoi, 61 Phan Chu Trinh, Hanoi, Vietnam; 7 Guangxi Institute of Cultural Relic Protection and Archaeology, Nanning, 530003 China; 8 Fujian Museum, Fuzhou, 350001 China; 9 Center for Austronesian Study, Jakarta, 12510 Indonesia; 10 The National Research Center for Archaeology, Jakarta, 12510 Indonesia; 11 Institute of History and Philology, Academia Sinica, Taipei, 11529 Taiwan; 12 Matzu Folk Culture Museum, Nangan, Lienchiang 20942 Taiwan; 13 Institute of Cultural Relics and Archaeology of Hunan, Changsha, 410008 China; 14 Institute of Cultural Relics and Archaeology of Zhejiang, Hangzhou, 310014 China; 15 Hemudu Site Museum, Ningbo, 315414 China; 16 Institute of Archaeology, Chinese Academy of Social Science, Beijing, 100710 China; 17 Henan Provincial Institute of Cultural Heritage and Archaeology, Zhengzhou, 450000 China; 18 Division of Tropical Health and Medicine, College of Medicine and Dentistry, James Cook University, Townsville, Queensland 4811 Australia; 19 Department of Anatomy, University of Otago, Dunedin, 9054 New Zealand; 20 Southern Institute of Social Sciences, Vietnam Academy of Social Science, 49 Nguyen Thi Minh Khai, Ho Chi Minh, Vietnam; 21 Commission for the Archaeology of Noneuropean Cultures of the German Archaeological Institute, 53173 Bonn, Germany

Correction to: *Scientific Reports* 10.1038/s41598-018-35426-z, published online 05 February 2019

In Figure 1, in the Historic/Modern Samples map, Cambodia, Celebes, Hainan, Laos, Papua New Guinea, Seman, South Moluccas and Vietnam are labelled incorrectly. The correct Figure 1 appears below.

**Figure 1:**
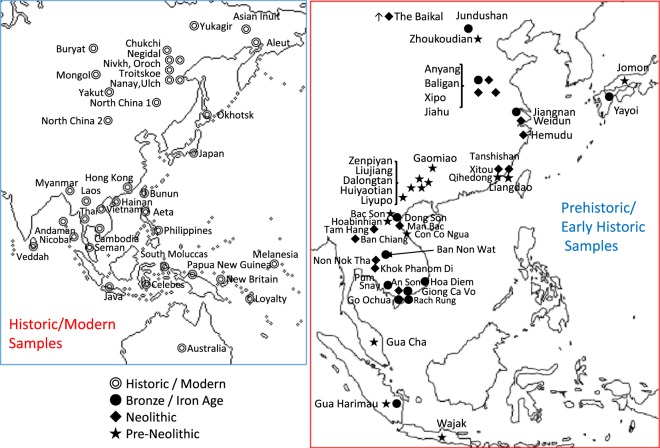
Map showing comparative sample localities.

